# Statistics of Medical Schools

**Published:** 1846-12

**Authors:** 


*Statistics of the Medical Schools of the United States for the Session* 1845–'46.—

| | Students. | Graduates. |
|---|---|---|
| University of Pennsylvania - - - | 462 | 168 |
| Transylvania University - - - | 171 | 58 |
| Medical Institution of Geneva College - | 179 | 39 |
| Medical Department Willoughby University | 164 | 30 |
| Albany Medical College - - - | 115 | 42 |
| Louisville Medical Institute - - - | 345 | 73 |
| Western Reserve College, (Cleveland) - | 161 | 52 |
| Jefferson Medical College - - - | 469 | 170 |
| Rush Medical College, (Chicago) - - | 50 | 9 |
| Harvard University, Boston, - - | 180 | 31 |
| College of Physicians and Surgeons, N.Y. | 219 | 38 |
| Med. Dep. University of the city of N.Y. | 425 | 131 |
| Ohio Medical College - - - - - | 192 | 46 |
| University of Maryland - - - | 147 | 40 |
| Medical College of Georgia - - - | 112 | 30 |
| Med. Dep. of Hampden Sidney College | 74 | 17 |
| Med. Dep. University of Missouri - - | 92 | 29 |
| Med. Dep. Illinois College - - - | | 13 |
| St. Louis University, - - - - - | 53 | 11 |
| Med. College of the State of S. Carolina | 210 | 74 |
| University of the City of N.Y. - - | 407 | 130 |
| Indiana Medical College, (Laporte) - | 71 | 17 |
| Berkshire Medical Institution - - - | 142 | 35 |
| Medical College of Louisiana - - - | 103 | 19 |
| Pennsylvania Medical College - - | 70 | 38 |
| Medical School of Maine, - - - - | 73 | 19 |
| Vermont Medical College, (Woodstock) | | 24 |
| Yale College - - - - - - - | 53 | 19 |
| Castleton Medical College - - - | 140 | 36 |

*American Jour. Med. Sci.*

